# Low-Dose Decitabine Augments the Activation and Anti-Tumor Immune Response of IFN-γ^+^ CD4^+^ T Cells Through Enhancing IκBα Degradation and NF-κB Activation

**DOI:** 10.3389/fcell.2021.647713

**Published:** 2021-03-15

**Authors:** Xiang Li, Liang Dong, Jiejie Liu, Chunmeng Wang, Yan Zhang, Qian Mei, Weidong Han, Ping Xie, Jing Nie

**Affiliations:** ^1^Department of Cell Biology, The Municipal Key Laboratory for Liver Protection and Regulation of Regeneration, Capital Medical University, Beijing, China; ^2^Department of Bio-therapeutic, The First Medical Center, Chinese PLA General Hospital, Beijing, China

**Keywords:** decitabine, CD4^+^ T cells, immune response, NF-κB activation, IκBα degradation

## Abstract

**Background:**

CD4^+^ T cells play multiple roles in controlling tumor growth and increasing IFN-γ^+^ T-helper 1 cell population could promote cell-mediated anti-tumor immune response. We have previously showed that low-dose DNA demethylating agent decitabine therapy promotes CD3^+^ T-cell proliferation and cytotoxicity; however, direct regulation of purified CD4^+^ T cells and the underlying mechanisms remain unclear.

**Methods:**

The effects of low-dose decitabine on sorted CD4^+^ T cells were detected both *in vitro* and *in vivo*. The activation, proliferation, intracellular cytokine production and cytolysis activity of CD4^+^ T cells were analyzed by FACS and DELFIA time-resolved fluorescence assays. *In vivo* ubiquitination assay was performed to assess protein degradation. Moreover, phosphor-p65 and IκBα levels were detected in sorted CD4^+^ T cells from solid tumor patients with decitabine-based therapy.

**Results:**

Low-dose decitabine treatment promoted the proliferation and activation of sorted CD4^+^ T cells, with increased frequency of IFN-γ^+^ Th1 subset and enhanced cytolytic activity *in vitro* and *in vivo*. NF-κB inhibitor, BAY 11-7082, suppressed decitabine-induced CD4^+^ T cell proliferation and IFN-γ production. In terms of mechanism, low-dose decitabine augmented the expression of E3 ligase β-TrCP, promoted the ubiquitination and degradation of IκBα and resulted in NF-κB activation. Notably, we observed that *in vitro* low-dose decitabine treatment induced NF-κB activation in CD4^+^ T cells from patients with a response to decitabine-primed chemotherapy rather than those without a response.

**Conclusion:**

These data suggest that low-dose decitabine potentiates CD4^+^ T cell anti-tumor immunity through enhancing IκBα degradation and therefore NF-κB activation and IFN-γ production.

## Introduction

Immunotherapy has become a standard approach for the treatment of some types of cancers and has the potential to control tumor development. Most immunotherapy strategies devote to reinvigorate T cell function to evoke effective anti-tumor immune responses (Borst et al., 2018) and most clinical settings focus on the exploiting of cytotoxic CD8^+^ T lymphocytes (CD8^+^ CTLs) (Rosenberg and Restifo, 2015; Ott et al., 2017). CD4^+^ T helper cells are recognized to be required for the formation of CD8^+^ CTLs. Currently, numerous studies have demonstrated that CD4^+^ T cells actively participate in shaping anti-tumor immunity (Kim and Cantor, 2014; Zander et al., 2019). Based on their functions and cytokine-producing patterns, CD4^+^ T helper cells are comprised of different functional subsets, including IFN-γ-producing T-helper 1 (Th1), T-helper 2 (Th2), T-helper 17 (Th17) and regulatory T cells (Tregs), which carry out specialized immunoregulatory functions to either enhance or inhibit immune response (Kim and Cantor, 2014). Th1 cells enhance CD8^+^ CTLs function and enable CD8^+^ CTLs to overcome the obstacles that typically hamper anti-tumor immunity, and Tregs are essential for maintenance of T cell homeostasis and prevention of autoimmunity. In addition, the cytotoxic effector CD4^+^ T cells, most closely related to Th1 subset, can directly eliminate tumor cells through the MHC class II-dependent manner, which destroy target cells by secreting granzyme B and perforin (Takeuchi and Saito, 2017). However, the functional diversity of CD4^+^ T cells and disorders of CD4^+^ T cell subsets weaken the anti-tumor responses (Lee et al., 2012). Thus, boosting Th1 and cytotoxic CD4^+^ T cell responses and inhibiting Tregs functions may obtain optimal anti-tumor responses.

Epigenetic modifying agent decitabine (5-Aza-2’-deoxycytidine, DAC) is a unique cytosine analog and an inhibitor of DNA methyltransferases (DNMTs) (Jones and Taylor, 1980). Decitabine has been approved for the treatment of hematological diseases, such as myelodysplastic syndrome (MDS) (Kantarjian et al., 2007) and acute myelogenous leukemia (AML) (Kelly et al., 2010). At the very beginning, decitabine was used as a chemotherapy drug with relatively high doses (Aparicio and Weber, 2002). Unfortunately, extreme cytotoxicity and myelosuppression limited the clinical applications of decitabine (Oki et al., 2007). Recently, preclinical investigations and clinical trials have shown that low doses of decitabine have minimal cytotoxicity and combination therapy could achieve an optimal anti-tumor effect (Mei et al., 2015; Yu et al., 2018). Genome-wide DNA methylation is a stable epigenetic characteristic in tumor cells and closely associates with tumor development and progression (Chatterjee et al., 2018). Moreover, emerging evidences have revealed that DNA hypermethylation impairs the immunogenicity and immune recognition, resulting in tumor immune escape (Maio et al., 2003). Low-dose decitabine suppresses function and induces degradation of DNMTs, considered as an immunotherapeutic drug to control tumor progression (Li et al., 2015; Yu et al., 2018). It is worth noting that most previous studies involved with the mechanism of low-dose decitabine therapy mainly focus on cancer cells and CD8^+^ T cells rather than CD4^+^ T cells (Li et al., 2015; Topper et al., 2020). Epigenetic modification plays an important role in the differentiation of CD4^+^ T cells (Shih et al., 2014; Tripathi and Lahesmaa, 2014), while the regulation of CD4^+^ T cells in anti-tumor activity still needs to be deeply explored.

In our previous clinical trials, we have demonstrated that low-dose decitabine treatment increases CD4/CD8 ratio among peripheral T cells (Nie et al., 2016) and CD4^+^ T cells infiltration in tumors. Furthermore, low-dose decitabine results in increased frequency of IFN-γ^+^ T cells (Li et al., 2017). However, the direct effect of low-dose decitabine on purified CD4^+^ T cells and regulation mechanisms remain unclear. In this study, we sorted CD4^+^ T cells, treated with low-dose decitabine, and detected the phenotype and cytolysis activity. Moreover, we investigated the mechanism of low-dose decitabine-mediated increased frequency of IFN-γ^+^ CD4^+^ T cells, and found that this effect was mediated by decitabine-induced protein degradation of IκBα, and this process was dependent on E3 ligase β-TrCP.

## Materials and Methods

### Human Peripheral Blood

The peripheral blood was obtained from the cancer patients enrolled in the clinical trials of Chinese PLA general hospital (www.clinicaltrials.gov: NCT01799083). The collection and storage of peripheral blood were consistent with our previous study (Li et al., 2017) and normal peripheral blood was obtained from healthy donors. All peripheral blood was collected with the informed consent as approved by the ethics committee of the Chinese PLA general hospital, Beijing, China.

### CD4^+^ T Cells Sorting, Culture, and Decitabine Treatment

Mouse CD4^+^ T cells were purified from splenocytes of C57BL/6J using CD4^+^ T Cell Isolation Kit (Miltenyi, Cat#130-104-454) according to the manufacturer’s guide. CD4^+^ T cells were activated *in vitro* with plate-bound 2 μg/ml anti-CD3 (Biolegend, Cat#100340) and 2 μg/ml anti-CD28 (Biolegend, Cat#102116) and recombinant IL-2 (cyagen, Cat#MEILP-0201) for 24 h. Human CD4^+^ T cells were isolated from peripheral blood mononuclear cells of healthy donors and cancer patients using CD4^+^ T cell Isolation (Miltenyi, Cat#130-045-101). The sorted human CD4^+^ T cells were activated with plate-bound anti-CD3 antibody (Takara, Cat#T210) and rIL-2 (cyagen, Cat#HEILP-0201) for 24 h. Activated CD4^+^ T cells were treated with PBS (CON) or decitabine (10 nM or indicated concentrations, Sigma-Aldrich, Cat#A3656) plus rIL-2 for 3 days. After decitabine treatment, CD4^+^ T cells were analyzed by flow cytometry or adoptively transferred into tumor-bearing mice.

### *In vitro* Th1 Differentiation

CD4^+^ T cells were sorted from mouse splenocytes and stimulated with plate-bound anti-CD3/CD28 in the presence of IL-12 (10 ng/mL) and anti-IL-4 (10 μg/ml) for 24 h. Activated T cells were treated with PBS or decitabine in the presence of IL-12 and anti-IL-4 for 3 days and flow cytometry was performed to detect the frequency of IFN-γ^+^ cells in CD4^+^ T cells.

### Flow Cytometry and Reagents

The following antibodies were purchased from Biolegend: CD3 PerCP (Cat#300326), CD4 APC (Cat#357408), CD4 PerCP (Cat#100432), Ki67 FITC (Cat#652410), Ki67 FITC (Cat#151212), IFN-γ PE (Cat#505808), IFN-γ BV421 (Cat#505830), IFN-γ FITC (Cat#502506), CD69 FITC (Cat#104506), CD28 PE (Ca#102106), CD25 APC (Cat#101910), T-bet PE (Cat#644812), CD45 BV510 (Cat#103138), CD107a PE (Cat#121612), Granzyme B FITC (Cat#515403), Perforin PE (Cat#154406), TNF-α APC (Cat#506308), and isotype-matched antibodies.

Surface marker staining was performed with mAbs for 15 min in PBS using indicated antibodies. For the intracellular cytokine expression detection, CD4^+^ T cells were stimulated with Cell Stimulation Cocktail (plus protein transport inhibitors) (eBioscience, Cat#00-4975-03) for 4 h prior to staining. For the *in vitro* co-culture assay, CD4^+^ T cells were co-cultured with colon cancer MC38 cells as the indicated ratio for 6 h, and intracellular protein transport inhibitor Brefeldin A (BFA, Beyotime, Cat#S1536) was added for 5 h before collection when assessing the intracellular proteins. To evaluate cell apopsis, freshly collected CD4^+^ T cells were processed into single-cell suspensions and stained with annexin V and 7-AAD according to the manufacturer’s instructions (BD, cat#559763). Cells were detected on DxFLEX (Beckman Coulter) and analyzed with the Kaluza Analysis 2.1 software (Beckman Coulter).

### CFSE Proliferation Assay

Purified CD4^+^ T cells were activated with *in vitro* plate-bound anti-CD3/CD28 and recombinant IL-2 for 24 h. CD4^+^ T cells were incubated at 37°C for 5 min with 2 μM CFSE diluted in PBS, and then an equal volume of cold FBS was used to stop the reaction. Subsequently, cells were washed twice with RPMI 1640 containing 10% FBS. Finally, CFSE-labeled CD4^+^ T cells were treated with PBS or decitabine for 3 days, and analyzed by flow cytometry.

### Cytotoxicity Assay

To detect the cytotoxicity of CD4^+^ T cells, DELFIA time-resolved fluorescence (TRF) assays were performed (PerkinElmer, Cat#AD0116). The processes of the staining, incubation and measure time-resolved fluorescence were operated according to the manufacturer’s instructions. Specific release represents the cytotoxic activity of CD4^+^ T cells. MC38 (mouse colon cancer cell line) or HCT116 (human colon cancer cell line) cells were used as targets to study the cytotoxicity of mouse or human CD4^+^ T cells, respectively.

### Inhibitors Treatment

CD4^+^ T cells were treated with respective five inhibitors, Ruxolitinib (Cat#S1378, 10 μM), Rapamicin (Cat#S1039, 100 nM), LY294002 (Cat# S1105, 10 μM), BAY 11-7082 (Cat#S2913, 10 μM), and ICG-001 (Cat#S2662, 5 μM) for 12 h followed by decitabine treatment. All inhibitors were purchased from Selleck.

### Quantitative Real-Time PCR (qRT-PCR)

Total RNA was isolated using TRIzol Reagent (ambion, Cat#15596018). Reverse transcription to cDNA was performed using RevertAid First Strand cDNA Synthesis Kit (Thermo Fisher Scientific, Cat#K1622). Real-time PCR was performed using SYBR Green Realtime PCR Master Mix (TOYOBO, Cat#QPK-201) and Applied Biosystems 7500 (life technologies). The following primers were used: IκBα, F, 5′-TGA AGGACGAGGAGTACGAGC-3′, R, 5′-TGCAGGAACGAGTC TCCGT-3′, β-TrCP: F, 5′-TCCCAAATGTGTCACTACCAGC-3′, R, 5′-AGTGCAGTTATGAAATCCCTCTG-3′, GAPDH, F, 5′-AACCTGCCAAGTATGATGA-3′, R, 5′-GGAGTTGCTGTT GAAGTC-3′.

### Western Blot and *in vivo* Ubiquitination Assays

T cells were collected and washed with cold PBS and then lysed in lysis buffer to isolated total protein. Nuclear protein extracts were isolated using a nuclear and cytoplasmic extraction kit (Thermo Fisher Scientific, Cat#78835). Protein extracts were quantified using BCA assays and equalized using the extraction reagent. The following antibodies were purchased from Cell Signaling Technology: primary antibodies against phosphor-IKKα/β Ser176/Ser177 (2697)/IKKβ (8943), phosphor-IκBα Ser32 (2859)/IκBα (9242), phosphor-p65 Ser536 (3031)/p65 (6956), β-TrCP (11984), β-actin (3700). Anti-SP1 antibody was purchased from Abcam (ab157123). The relevant secondary antibodies were performed. All the antibodies were purchased from Cell Signaling Technology.

For *in vivo* ubiquitination assay, sorted CD4^+^ T cells were treated with MG132 (50μM, Sigma-Aldrich, Cat#M7449) before collection, and cells were lysed in modified RIPA lysis buffer [10 mM Tris-HCl (pH 7.5), 150 mM NaCl, 5 mM EDTA, 1% (v/v) NP-40, 1% sodium deoxycholate, 0.025% SDS, 1 × protease inhibitors]. The lysates were immunoprecipitated with anti-IκBα antibody and detected by western blot.

### Animal Experiments, Adoptive T Cell Transfer, and Tumor Digestion

Six to eight weeks old Balb/c nude mice were purchased from the SPF Biotechnology Co., Ltd. (Beijing, China). All mice were housed under pathogen-free conditions and all animal experiments were performed under protocols approved by Scientific Investigation Board of Chinese PLA General Hospital, Beijing, China. Mouse colon cancer MC38 cells were cultured with RPMI-1640 medium containing 10% fetal bovine serum and 1% penicillin and streptomycin. MC38 cells (1 × 10^5^) were harvested and wished twice with PBS then implanted subcutaneously into the right flank.

The tumor-bearing animals received intravenous injection of PBS or decitabine-treated CD4^+^ T cells (1 × 10^6^ per mouse) when tumor volumes reached a size of around 100 mm^3^. Tumor size was measured every 3 days in two dimensions by caliper. The tumor volume was calculated according to the formula (length × width^2^)/2 and mice were sacrificed at the indicated time points or when tumor volume reached >1.5 cm^3^ (endpoint). Survival analysis was performed for mice that received decitabine or PBS-treated CD4^+^ T cells transfer.

On the indicated days, tumors were excised, manually dissociated and digested with Tumor Dissociation Kit for 1 h (Miltenyi, Cat#130-096-730), followed by mashing through 70 μm nylon cell strainer. Cells were harvested and washed twice with PBS then cells were resuspended in PBS for further detection.

### Statistical Analysis

Data are presented as the mean ± S.D. of at least three independent experiments. Statistical comparisons between experimental groups were analyzed using the Student *t*-test. Kaplan-Meier survival analysis was performed to estimate the survival curves for tumor-bearing mice that received PBS or decitabine-treated CD4^+^ T cells transfer and significant differences were evaluated by a log-rank test. A two-way repeated-measures analysis of variance (ANOVA) was conducted to evaluate the effect of time-group interaction. Statistical analysis was performed using GraphPad Prism software 8.0. A two-tailed *p* < 0.05 was considered statistically significant.

## Results

### Low-Dose Decitabine Promotes the Activation and Proliferation of Sorted CD4^+^ T Cells

**FIGURE 1:**
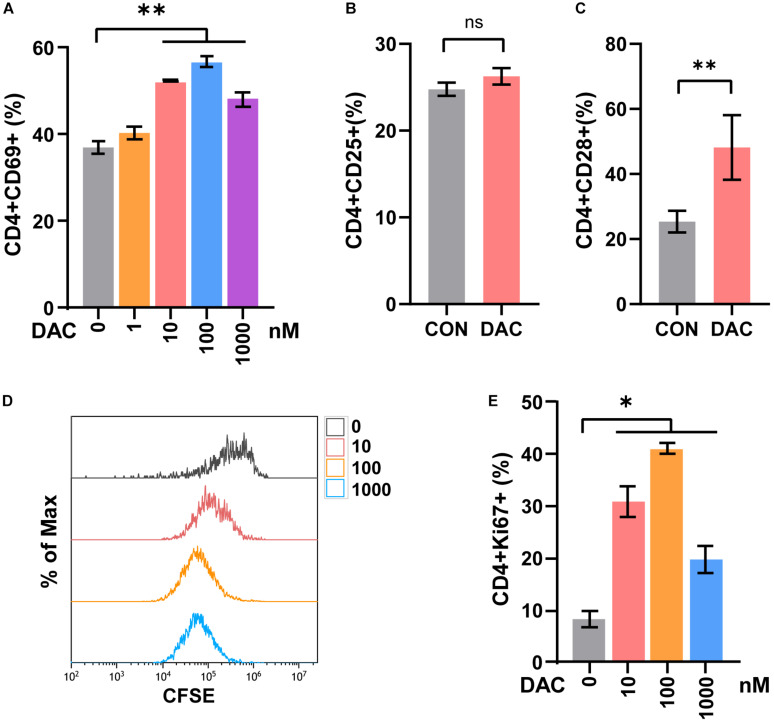
Low-dose decitabine promotes the activation and viability of sorted CD4^+^ T cells *in vitro*. **(A)** To detect T cell early activation markers, mouse CD4^+^ T cells were sorted from splenocytes, activated with immobilized anti-CD3/CD28 for 24 h, and treated with different concentrations of decitabine (DAC, 0, 1, 10, 100, and 1,000 nM) plus IL-2 for 2 days. The surface marker CD69 was detected by flow cytometry. **(B,C)** After anti-CD3/CD28 activation, CD4^+^ T cells were treated with PBS (CON) or 10 nM DAC for 3 days, the expressions of CD25 **(B)** and CD28 **(C)** were detected by flow cytometry. **(D)** Sorted CD4^+^ T cells were activated with anti-CD3/CD28, the CFSE-labeled (2 μM) T cells were then treated with PBS or different concentrations of decitabine for 3 days, and detected by flow cytometry. **(E)** Ki67 was detected by flow cytometry on day three after PBS or decitabine treatment. All results from three independent experiments are shown. ^∗^*P <*0.05 and ^∗∗^*P <* 0.01. ns, not significant.

Previous studies demonstrated that low-dose decitabine (10 nM) has anti-tumor activity in solid tumors *in vivo* (Tsai et al., 2012; Li et al., 2017). To investigate the direct effect of low-dose decitabine on CD4^+^ T cell activation, mouse CD4^+^ T cells were sorted from splenocytes, activated with immobilized anti-CD3/CD28, and treated with different concentrations of decitabine (0, 1, 10, 100, and 1,000 nM). We observed a significant increase in CD69^+^ cells as a percentage of CD4^+^ cells following decitabine treatment at a concentration of 10 nM or higher (100 and 1,000 nM), indicating that 10 nM low-dose of decitabine could enhance the activation of purified CD4^+^ T cells (Figure 1A). The ratio of CD25^+^ cells in CD4^+^ T cells was not changed with 10 nM decitabine treatment (Figure 1B). Moreover, we examined the expression of CD28, a co-stimulatory molecule, which played a pivotal role in triggering CD4^+^ T cell activation. The results showed that 10 nM of low-dose decitabine markedly increased the percentage of CD28^+^CD4^+^ T cells (Figure 1C). We further detected the proliferation capacity of CD4^+^ T cells in response to decitabine. The CD4^+^ T cells were activated by anti-CD3/CD28, and the CFSE labeled CD4^+^ T cells were administrated with PBS or decitabine for 3 days and analyzed by flow cytometry. As shown in Figure 1D, low-dose decitabine enhanced the proliferation of CD4^+^ T cells (Figure 1D). The upregulated expression of Ki67 further confirmed that 10 nM of low-dose decitabine mediated proliferation of activated CD4^+^ T cells, and we noticed that the ratio of Ki67^+^ cells was reduced with decitabine at a relative higher concentration of 1,000 nM as compared to that with lower dose of 10 nM (Figure 1E). These results suggested that low-dose decitabine treatment promoted the activation and proliferation of CD4^+^ T cells.

### Low-Dose Decitabine Increases the Frequency of Th1 Subset and CD4^+^ T Cell Cytolysis Activity

**FIGURE 2:**
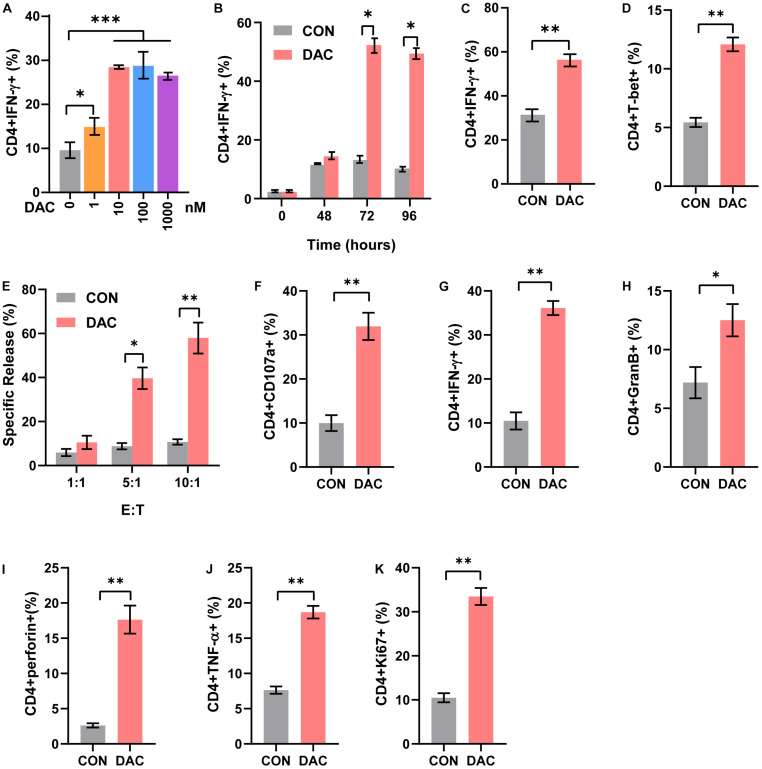
Low-dose decitabine increases the frequency of Th1 subset and CD4^+^ T cell cytolysis activity. **(A)** Purified CD4^+^ T cells were activated with immobilized anti-CD3/CD28, treated with PBS or decitabine (1, 10, 100, 1,000 nM) for 3 days, and IFN-γ expression was detected by flow cytometry. **(B)** Purified CD4^+^ T cells were activated with immobilized anti-CD3/CD28, treated with PBS or 10 nM decitabine for the indicated times, and IFN-γ was detected by flow cytometry. **(C)** Sorted CD4^+^ T cells were treated under Th1 polarization conditions, with PBS or 10 nM decitabine treatment for 3 days, and IFN-γ was detected by flow cytometry. **(D)** T-bet was detected by flow cytometry in purified CD4^+^ T cells after PBS or 10 nM decitabine treatment for 3 days followed by anti-CD3/CD28 stimulation. **(E)** Sorted CD4^+^ T cells were activated by anti-CD3/CD28, and treated with PBS or 10 nM decitabine for 3 days. The cytotoxicity of CD4^+^ T cells was assessed by DELFIA time-resolved fluorescence (TRF) assays against MC38 cells at different E:T ratios as indicated. **(F–K)** PBS- or 10 nM decitabine-pretreated CD4^+^ T cells were cocultured with MC38 cells for 24 h, and CD107a **(F)**, IFN-γ **(G)**, granzyme B **(H)**, perforin **(I)**, TNF-α **(J)** and Ki67 **(K)** levels were detected by flow cytometry. The values are three independent experiments. ^∗^*P <* 0.05, ^∗∗^*P <* 0.01, ^∗∗∗^*P <* 0.001.

CD4^+^ T cells include distinct functional subsets based on their function and cytokine secretion patterns, such as Th1, Th2, Th17, and Tregs (Kim and Cantor, 2014). We next explored the effect of low-dose decitabine on CD4^+^ T cell subsets. Purified CD4^+^ T cells were treated with different concentrations of decitabine for 3 days. The flow cytometry assay showed that the frequency of Th1 subset, which produced IFN-γ, was increased following 10 nM of low-dose decitabine treatment (Figure 2A). Notably, low-dose decitabine-induced Th1 cell expansion was not a transient action and required an exposure longer than 2 days, consistently with the slow and memory response of low doses of decitabine *in vivo* (Figure 2B). Moreover, under *in vitro* Th1 polarization conditions, treatment of low-dose decitabine markedly increased the frequency of IFN-γ^+^ cells in CD4^+^ T cells (Figure 2C). As expected, the expression of T-bet was increased after low-dose decitabine treatment (Figure 2D), which was a key transcription factor associated with Th1 differentiation (Pritchard et al., 2019; Xia et al., 2019).

The cytotoxic CD4^+^ T cells played important roles in anti-tumor immunity and were reported to be a close relative of Th1 cells. To investigate whether low-dose decitabine affects the cytotoxicity of CD4^+^ cells, we performed the DELFIA time-resolved fluorescence (TRF) assays. The results showed that low-dose decitabine-treated CD4^+^ T cells exhibited an elevated cytotoxicity against mouse colon carcinoma MC38 cells as compared with control CD4^+^ T cells (Figure 2E). Being a marker of cytotoxic T cell degranulation, CD107a, was markedly enhanced on the surface of CD4^+^ T cells with low-dose decitabine pretreatment (Figure 2F). Consistently, low-dose decitabine promoted the expression of IFN-γ, granzyme B, perforin and TNF-α on CD4^+^ T cells when co-cultured with MC38 cells (Figures 2G–J). Furthermore, the proliferating potential of CD4^+^ T cells was also increased with decitabine treatment (Figure 2K). Taken together, low-dose decitabine could potentiate the cytolysis activity of CD4^+^ T cells *in vitro*.

### Low-Dose Decitabine-Pretreated CD4^+^ T Cells Inhibits Tumor Growth *in vivo*

**FIGURE 3:**
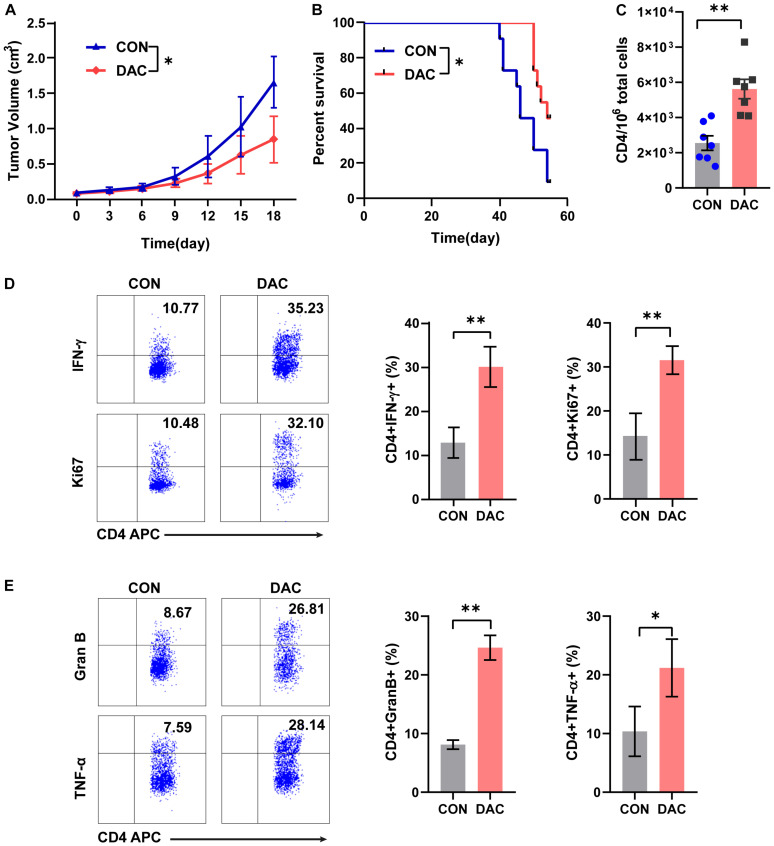
Low-dose decitabine-treated CD4^+^ T cells inhibit tumor growth *in vivo.* Subcutaneously implantation of MC38 cells received PBS- or 10 nM decitabine-treated CD4^+^ T cells when the tumor volume reached 100 mm^3^. **(A)** The tumor volume was measured every 3 days (*n* = 6/group). **(B)** Survival cures of MC38-bearing mice received PBS- or 10 nM decitabine-treated CD4^+^ T cells (*n* = 11/group). **(C–E)** The MC38-bearing mice were sacrificed on day 18 after CD4^+^ T cell transfer, the absolute number of tumor infiltrated CD4^+^ T cells were assessed **(C)**; and percentages of IFN-γ^+^, Ki67^+^ **(D)**, granzyme B^+^ and TNF-α^+^ **(E)** cells among tumor infiltrated CD4^+^ T cells were detected by flow cytometry. All results from three independent experiments are shown. ^∗^*P <* 0.05. ^∗∗^*P <* 0.01.

To further study the anti-tumor capacity of low-dose decitabine-pretreated CD4^+^ T cells *in vivo*, we performed a tumor-bearing xenograft model of mouse colon cancer MC38 cells and transferred low-dose decitabine-pretreated CD4^+^ T cells. The results showed that infusion of low-dose decitabine-pretreated CD4^+^ T cells significantly inhibited tumor growth and prolonged survival as compared to control CD4^+^ T cells (Figures 3A,B). Importantly, higher number of infiltrated CD4^+^ T cells was observed in tumors treated with decitabine-primed CD4^+^ T cells (Figure 3C). Moreover, the frequencies of IFN-γ^+^ and Ki67^+^ cells as in tumor infiltrated CD4^+^ T cells were dramatically increased in mice transferring low-dose decitabine-pretreated CD4^+^ T cells comparing transferring control CD4^+^ T cells (Figure 3D). In these mice received low-dose decitabine-pretreated CD4^+^ T cells, the expression levels of cytotoxic marker granzyme B and TNF-α were also upregulated in tumor infiltrated CD4^+^ T cells (Figure 3E). Therefore, low-dose decitabine treated CD4^+^ T cells had improved anti-tumor activity *in vivo*.

### Low-Dose Decitabine Promotes CD4^+^ T Cell Anti-Tumor Immune Response Dependent on NF-κB Signaling

**FIGURE 4:**
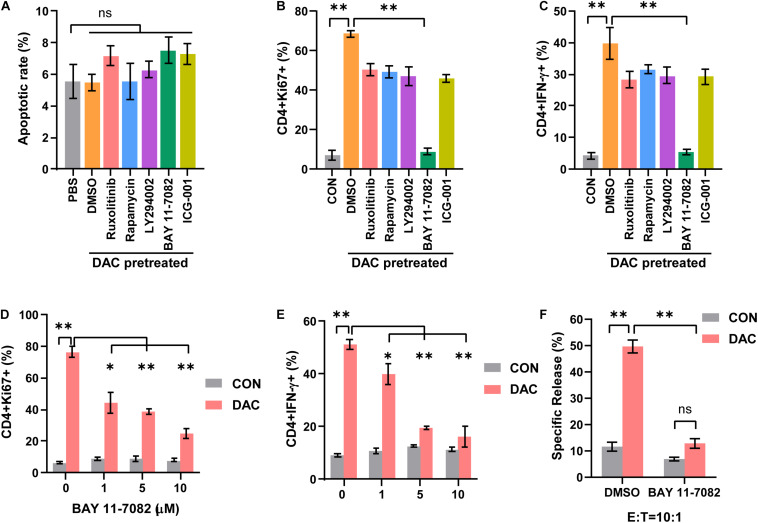
Low-dose decitabine promotes CD4^+^ T cell-mediated immune response through NF-κB signaling. **(A–C)** Activated CD4^+^ T cells were treated with PBS or 10 nM decitabine for 3 days, the decitabine-pretreated cells further received DMSO, Ruxolitinib (10 μM), Rapamycin (100 nM), Ly294002 (10 μM), BAY 11-7082 (10 μM), or ICG-001 (5 μM) for 12 h. Cells were collected, and cell apoptosis **(A)**, Ki67 **(B)**, and IFN-γ **(C)** levels were tested by flow cytometry. **(D,E)** Activated CD4^+^ T cells were pretreated with PBS or 10 nM decitabine for 3 days, these cells received treatment with the indicated dose of BAY 11-7082 for 12 h, and Ki67 **(D)** and IFN-γ **(E)** levels were detected by flow cytometry. **(F)** PBS or 10 nM decitabine-pretreated CD4^+^ T cells were treated with DMSO or BAY 11-7082 (10 μM) for 12 h, the cytotoxicity against MC38 cells was examined by using DELFIA time-resolved fluorescence (TRF) assays. All results from three independent experiments are shown. ^∗^*P <* 0.05. ^∗∗^*P <* 0.01.

In order to investigate the mechanism underlying low-dose decitabine-induced CD4^+^ T cell proliferation and function, we explored the upstream signaling pathway that controlled the viability of CD4^+^ T cells. Inhibitors targeting JAK1/2 (ruxolitinib), mTOR (rapamycin), PI3K (LY294002), NF-κB (BAY 11-7082) and Wnt/β-catenin (ICG-001) signaling were used. First, low-dose decitabine treatment had minimal effect on CD4^+^ T cell apoptosis, and all these inhibitors did not result in increased apoptosis in low-dose decitabine pretreated-CD4^+^ T cells (Figure 4A). Interestingly, we noticed that addition of NF-κB inhibitor markedly suppressed decitabine-induced proliferative capacity and IFN-γ secretion in CD4^+^ T cells (Figures 4B,C). Results confirmed that NF-κB inhibitor attenuated decitabine-mediated CD4^+^ T cell viability and IFN-γ expression in a dose-dependent manner (Figures 4D,E). Moreover, we observed that NF-κB inhibitor decreased the cytotoxicity of decitabine-treated CD4^+^ T cells against colon cancer MC38 cells (Figure 4F).

### Low-Dose Decitabine Reinforces NF-κB Activation by Promoting IκBα Ubiquitination and Degradation

**FIGURE 5:**
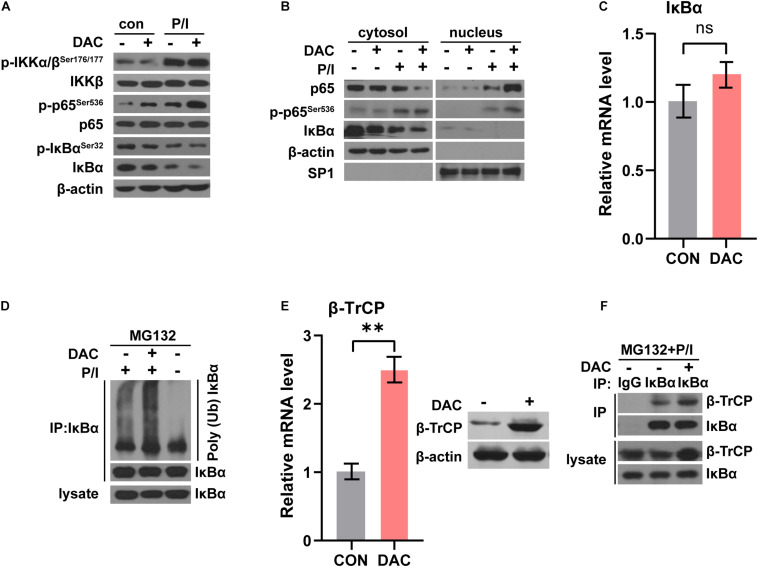
Low-dose decitabine enhances NF-κB activation by promoting IκBα ubiquitination and degradation. **(A)** PBS or 10 nM decitabine-pretreated CD4^+^ T cells were stimulated with or without PMA/Ionomycin (P/I) for 15 min. The total proteins were extracted and the expression levels of the indicated proteins were detected by western blot. **(B)** Cytosolic and nuclear fractions were extracted from PBS or 10 nM decitabine-pretreated CD4^+^ T cells with or without PMA/Ionomycin stimulation, and the indicated proteins were detected by western blot. **(C)** The mRNA level of IκBα in CD4^+^ T cells with or without 3 day 10 nM decitabine treatment was detected by qRT-PCR assay. **(D)** PBS or 10 nM decitabine-primed CD4^+^ T cells were treated with the protein degradation inhibitor MG132 (20 μM) for 8 h before collecting, and stimulated with or without 15 min PMA/Ionomycin before collecting as indicated. Cells were immunoprecipitated with anti-IκBα antibody, and both lysate and immunoprecipitate were analyzed by western blot with the indicated antibodies. **(E)** The mRNA and protein level of β-TrCP in CD4^+^ T cells with or without 3 day 10 nM decitabine treatment was detected by qRT-PCR assay and western blot. **(F)** PBS or 10 nM decitabine-primed CD4^+^ T cells were treated with the proteasome degradation inhibitor MG132 (20 μM) for 8 h before collecting, and stimulated with 15 min PMA/Ionomycin before collecting. Cells were immunoprecipitated with anti-IgG or anti-IκBα antibody as indicated, and both lysate and immunoprecipitate were analyzed by western blot. P/I, PMA/Ionomycin. ^∗∗^*P <* 0.01.

NF-κB signaling was crucial and activated in T cells following anti-CD3 stimulation (Gerondakis et al., 2014). In canonical NF-κB signaling pathway, the phosphorylation and ubiquitination of IκBα results in IκBα degradation, which allows NF-κB p50/p65 heterodimer nucleus translocation. NF-κB activation was characterized by the detection of p65 nuclear staining and phosphorylation of p65 at Ser536 (Gerondakis et al., 2014). We next assessed the influence of low-dose decitabine on NF-κB activation in CD4^+^ T cells. As shown in Figure 5A, PMA/Ionomycin (P/I) stimulation induced NF-κB activation as the increased phosphorylation of IKKα/β, p65 and downregulation of IκBα in sorted CD4^+^ T cells. We found that low-dose decitabine-primed CD4^+^ T cells might display more robust NF-κB activation upon P/I stimulation as compared to control CD4^+^ T cells, since the higher level of phospho-p65 (Figure 5A). We next prepared nuclear and cytoplasmic fractions from PBS or decitabine-primed CD4^+^ T cells treated with or without PMA/Ionomycin. Low-dose decitabine pretreated CD4^+^ T cells revealed dramatic translocation of p65 from the cytosol to the nucleus and diminished IκBα level in response to P/I stimulation, further confirming the enhanced activation of NF-κB (Figure 5B). In these cells, we noticed that the level of phosphor-IKKα/β was minimal elevated, and IκBα expression was reduced, suggesting that the increased IκBα degradation might trigger NF-κB activation.

To test this possibility, we first detected IκBα mRNA level and the qRT-PCR assay showed that the mRNA level of IκBα was not changed with low-dose decitabine treatment (Figure 5C). The *in vivo* ubiquitination assay demonstrated that IκBα ubiquitination was significantly enhanced in decitabine-pretreated CD4^+^ T cells with P/I stimulation as compared to control CD4^+^ T cells (Figure 5D). IκBα is degraded followed by polyubiquitination by the SCF^β–*TrCP*^ complex, among which β-TrCP associates with IκBα for ubiquitination (Winston et al., 1999). It has been reported that low expression of β-TrCP was associated with promoter hypermethylation and decitabine treatment restored mRNA and protein expression of β-TrCP with demethylation at promoter region (Tseng et al., 2008). Similarly, we observed that both the mRNA and protein levels of β-TrCP were increased after low-dose decitabine treatment in CD4^+^ T cells (Figure 5E). Therefore, the protein interaction between β-TrCP and IκBα was boosted in decitabine-primed CD4^+^ T cells (Figure 5F). These results suggest that low-dose decitabine potentiates NF-κB activation in CD4^+^ T cells by enhancing β-TrCP expression and mediated IκBα degradation.

### Low-Dose Decitabine Therapy Enhances NF-κB Activation in Human CD4^+^ T Cells and Associates With Clinical Response in Solid Tumor Patients

**FIGURE 6:**
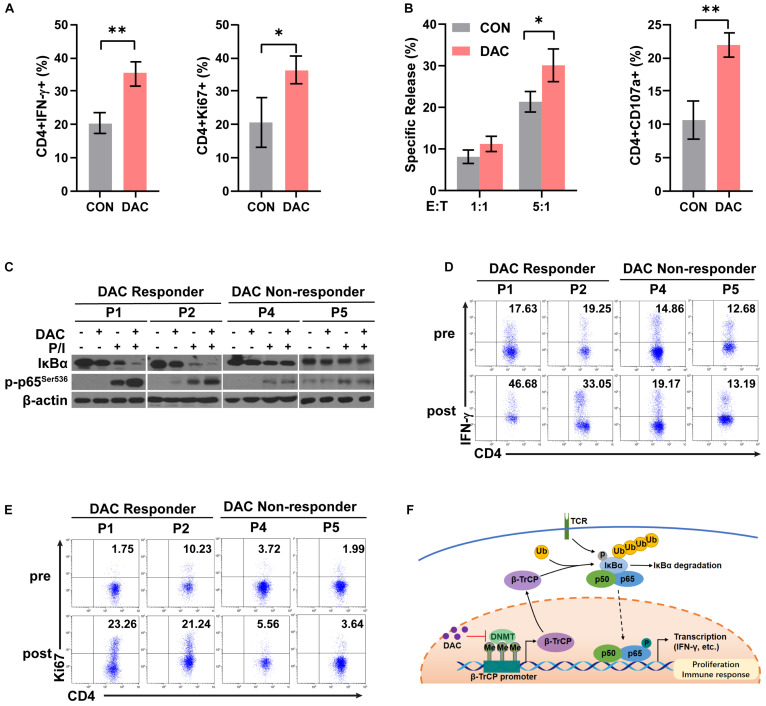
Low-dose decitabine therapy enhances NF-κB activation in CD4^+^ T cells and associates with clinical response in solid tumor patients. **(A,B)** Human CD4^+^ T cells were sorted from peripheral blood mononuclear cells of healthy donors, stimulated with anti-CD3 for 24 h, and treated with PBS or 10 nM decitabine for 3 days. After PBS or decitabine treatment, IFN-γ and Ki67 levels were detected by flow cytometry **(A)**. PBS or decitabine-pretreated CD4^+^ T cells were co-cultured with HCT116 cells, the cytotoxicity of human CD4^+^ T cells against HCT116 cells was determined by using DELFIA time-resolved fluorescence (TRF) assays at the indicated E:T ratios, and CD107a level was detected by flow cytometry **(B)**. **(C)** CD4^+^ T cells were sorted from solid tumor patients (P1, P2, P4, and P5) before treatment. Cells were treated with PBS or 10 nM decitabine for 3 days followed by 24 h-anti-CD3 antibody activation, and stimulated with PMA/Ionomycin for 15 min before collecting. The total proteins were extracted and the expression levels of the indicated proteins were detected by western blot. **(D,E)** CD4^+^ T cells were sorted from solid tumor patients (P1, P2, P4, and P5) before and after 5 day decitabine therapy, the frequencies of IFN-γ^+^ cells or Ki67^+^ cells as in CD4^+^ T cells were analyzed using flow cytometry. All results from three independent experiments are shown. ^∗^*P <* 0.05. **(F)** The proposed model for decitabine-mediated NF-κB activation and immune response. ^∗∗^*P <* 0.01.

To further examine the effect of low-dose decitabine on human CD4^+^ T cells, purified CD4^+^ T cells were prepared from peripheral blood of healthy donors, activated by using anti-CD3 antibody plus IL2 for 24 h, and treated with or without 10 nM decitabine for 3 days. The frequencies of IFN-γ^+^ and Ki67^+^ cells were increased after *in vitro* decitabine treatment (Figure 6A). In addition, low-dose decitabine enhanced the cytotoxicity of CD4^+^ T cells against colon cancer HCT116 cells, and increased CD107a expression (Figure 6B). To further explore whether decitabine-mediated NF-κB activation in CD4^+^ T cells plays an important role with decitabine-based therapy in solid tumor patients, we sorted peripheral CD4^+^ T cells from patients before treatment. We observed that in patients (P1 and P2) who had a response to decitabine-based therapy, *in vitro* low-dose decitabine pretreatment promoted IκBα degradation and increased p65 phosphorylation with P/I stimulation in CD4^+^ T cells; while in patients (P4 and P5) who did not acquired a response to decitabine-based therapy, *in vitro* decitabine pretreatment displayed no ability to enhance NF-κB activity (Figure 6C). Moreover, low-dose decitabine treatment increased IFN-γ production and Ki67 level in CD4^+^ T cells from decitabine-based therapy responders P1 and P2 rather than non-responders P4 and P5 (Figures 6D,E). These results suggested that low-dose decitabine therapy augmented CD4^+^ T cell immune response in solid tumor patients by promoting NF-κB transcription activation and thus inflammatory cytokine IFN-γ secretion, which was achieved by the upregulation of IκBα E3 ligase β-TrCP and the enhanced ubiquitination and degradation of IκBα (Figure 6F).

## Discussion

DNA methylation represents an important layer of silencing of gene expression and plays important roles in various biological processes. Decitabine, a DNA methyltransferase inhibitor, was effective in treating hematological tumors, such as MDS and AML. It has been widely investigated that genome-wide DNA promoter demethylation occurred in tumor cells after decitabine treatment, resulting in an increased immunogenicity and immune recognition (Li et al., 2015; Topper et al., 2020). However, the anti-tumor mechanisms of the epigenetic modifying agents were not fully clarified.

Besides cancer cells, the effect of epigenetic modulation on T cells has been concerned in recent years. As the main cytotoxic effector cell, decitabine-mediated reprogramming of CD8^+^ T cells were extensively explored, and emerging evidence has demonstrated that DNA methylation was directly involved in CD8^+^ T cell differentiation and function (Chiappinelli et al., 2016; Ghoneim et al., 2017; Henning et al., 2018). Our previous study reported that low-dose decitabine promoted the activation and viability of IFN-γ^+^ T cells in CD3^+^ T cells (Li et al., 2017). Herein, we focused on the regulation of purified CD4^+^ T cells by decitabine, and demonstrated that 10 nM of low-dose decitabine enhanced CD4^+^ T cell proliferation and activation, especially the IFN-γ^+^CD4^+^ T cells. The epigenetic modifying agents might play distinct roles with different concentrations and in diverse models. Decitabine induced FOXP3 expression in CD4^+^CD25^–^ T cells at a concentration of 1 μM or higher, and could convert the non-Tregs into Tregs with suppressor functions (Choi et al., 2010). While 1 μM of decitabine treatment impeded the proliferation of CD4^+^CD25^*high*^FOXP3^+^ Tregs and fostered Th1 polarization in Tregs (Landman et al., 2018). In an experimental autoimmune encephalomyelitis mouse model characterized by autoreactive T cells and dysregulated innate immune response, Wang et al. found that long-term higher-dose of decitabine treatment inhibited T cell proliferation due to the increased expression of TET2 and cell cycle inhibitors (Wang et al., 2017). We also identified low-dose decitabine directly increased the cytotoxic activity of CD4^+^ T cells against tumor cells *in vitro*, which might be due to the elevated frequency of IFN-γ^+^ CD4^+^ T subset following decitabine treatment. However, since the lack of antigen-specific CD4^+^ T cells, we could not determine whether low-dose decitabine promoted the activation and cytolysis capacity of cytotoxic CD4^+^ T cells.

Low-dose decitabine induced upregulation of a series of genes. In addition to direct DNA demethylation-mediated gene activation, the epigenetic agents could also affect the action of protein posttranslational modifications. In the canonical NF-κB signaling pathway, ubiquitination and degradation of IκBα leads to NF-κB translocation to the nucleus and transcription activation of a series of genes in immune response and proliferation (Hayden and Ghosh, 2011). NF-κB pathway regulates T cell differentiation and development through TCR and CD28 activation (Oh and Ghosh, 2013). In this study, we observed that low-dose decitabine treatment augments the expression of E3 ligase β-TrCP, which potentiates the degradation of IκBα, and thus enhancing the activation of NF-κB. The β-TrCP/IκBα/NF-κB pathway provided a new regulation pattern of NF-κB signaling in T cells. Moreover, NF-κB was aberrantly activated in most tumor cells, and we did not detect further NF-κB activation in tumor cells (data not shown), suggesting the distinct regulation of epigenetic agents in different types of cells.

Besides the hematological malignancies, low-dose decitabine therapy had efficacy in some solid tumors. Decitabine pretreatment could increase the sensitivity of cancer cells to chemotherapy or targeted therapy in a part of patients. No or weak association between decitabine response and DNA methylation status of specific genes were reported (Fandy et al., 2009). We previously reported that response to decitabine-primed chemotherapy might be related to the increased frequency of IFN-γ^+^ T cells in solid tumor patients (Li et al., 2017). In the current study, we demonstrated that low-dose decitabine-induced activation of IFN-γ^+^CD4^+^ T cell subset was dependent on the enhancement of NF-κB pathway. Moreover, we found that the NF-κB signaling was activated in response to *in vitro* decitabine treatment in CD4^+^ T cells from responsive patients, which was related to an increased frequency of peripheral IFN-γ^+^CD4^+^ T cells following *in vivo* decitabine therapy in ovarian cancer patients, further confirming the IκBα/NF-κB/IFN-γ regulation mechanism by low-dose decitabine in CD4^+^ T cells. However, due to limited clinical samples, the levels of β-TrCP in CD4^+^ T cells from responder and non-responder patients with decitabine therapy were still unclear. Whether the regulation of NF-κB pathway in CD4^+^ T cells played an essential role in producing anti-tumor response to decitabine-primed chemotherapy in solid tumor patients, and exploring the potential role of β-TrCP as a biomarker in decitabine-mediated CD4^+^ T-cell activation in patients is worthy of our further investigation.

## Conclusion

In conclusion, our study demonstrates that low-dose decitabine induces degradation of IκBα by β-TrCP through a proteasome degradation pathway, to boost NF-κB activation and thus promotes the proliferation of IFN-γ^+^CD4^+^ T cells and enhances the anti-tumor immune activity of CD4^+^ T cells (Figure 6F).

## Data Availability Statement

The original contributions presented in the study are included in the article, further inquiries can be directed to the corresponding authors.

## Ethics Statement

The studies involving human participants were reviewed and approved by the Ethics Committee of the Chinese PLA general hospital. The patients/participants provided their written informed consent to participate in this study. The animal study was reviewed and approved by the Ethics Committee of the Chinese PLA general hospital.

## Author Contributions

XL and LD performed experiments and analyzed data. JL and QM acquired data. CW and YZ analyzed clinical data. PX and WH given material support and reviewed the manuscript. JN designed the project and wrote the manuscript. All authors contributed to the article and approved the submitted version.

## Conflict of Interest

The authors declare that the research was conducted in the absence of any commercial or financial relationships that could be construed as a potential conflict of interest.
